# Network and Gene Set Enrichment Analysis of Adipokine Drivers of Prostate Cancer; Unravelling the Mechanistic Link Between Excess Adiposity and Prostate Cancer Risk

**DOI:** 10.1002/cam4.71468

**Published:** 2025-12-26

**Authors:** Zachary Dovey, Elena Tomas Bort, Jeffrey I. Mechanick

**Affiliations:** ^1^ General Urology, Robotics and Urologic Oncology, Department of Urology Mount Sinai Queens and Icahn School of Medicine at Mount Sinai New York New York USA; ^2^ Centre for Technology and Bioinformatics Millars Almazora Spain; ^3^ Kravis Center for Clinical Cardiovascular Health at Mount Sinai Fuster Heart Hospital Icahn School of Medicine at Mount Sinai New York New York USA

**Keywords:** adipose based chronic disease, inflammation, lifestyle, network analysis, obesity, oncogenesis, prostate cancer

## Abstract

**Background:**

Adiposity‐Based Chronic Disease (ABCD), a novel model housing obesity, insulin resistance, and adipokine‐related inflammation, increases the risk of aggressive prostate cancer (PCa), posttreatment PCa recurrence, and PCa mortality. This paper provides a new network analysis of relevant metabolic drivers to provide insight into the ABCD–PCa relationship.

**Methods:**

A literature search was performed using the terms “prostate cancer” AND “obesity” AND “inflammation”, with 629 references found, from which 17 reviews were chosen. Biomarkers identified from these reviews were characterized by cellular origin, signaling pathway, and oncogenic effect. The Webgestalt gene analysis toolkit was then used to generate modular‐based network analyses and gene ontology (GO) categories of these biomarkers for interpretation.

**Results:**

14 prominent biomarkers were identified influencing PCa risk through cellular proliferation, resisting cell death, metabolic reprogramming, tumor‐promoting inflammation, avoiding immune destruction, angiogenesis, and activating invasion. Network analyses of biomarker interactions highlighted prominent roles of monocyte chemoattractant protein‐1, interleukin‐1β, and C‐X‐C motif chemokine ligand 1. Top GO categories for the wider ABCD‐PCa network found key roles of ABCD‐gut microbiome dysbiosis and exposure of periprostatic white adipose tissue to the prostate microbiome (involving bacterial and lipopolysaccharide‐induced inflammation).

**Conclusion:**

Top hypotheses to guide molecular targeted therapies and lifestyle biomarker panels for PCa in ABCD relate to MCP‐1, IL‐1β, and CXCL1 signaling, as well as gut microbiome dysbiosis and the exposure of the periprostatic adipose tissue to the prostate microbiome. Further research and possible clinical trials allowing histological examination of pre‐ and post‐lifestyle intervention PCa tissue may provide further insights.

**Abbreviations**
ABCD: adiposity‐based chronic disease
AMPK: adenosine monophosphate (AMP) activated protein kinase
AP‐1: activator protein 1
AR: androgen receptor
ASC: adipose stromal cell
CCL: “CC” motif chemokine ligand (CC motif refers to the position of two adjacent cysteine residues in the final protein)
CXCL: CXC‐chemokine ligand (CXC refers to the position of two cysteine residues near the terminal of the protein, with any other amino acid representing X in the final protein)
DOCK2: dedicator of cytokinesis 2
ECM: extracellular matrix
EMT: epithelial mesenchymal transition
ERK: extracellular signal regulated kinase
FAK: focal adhesion kinase
FGF: fibroblast growth factor
JAK: Janus kinase
JNK: c‐Jun proto‐oncogene N‐terminal kinase
MAPK: mitogen‐activated protein kinase
MEK: mitogen‐activated protein kinase (MAPK)/extracellular signal‐regulated kinase (ERK) kinase
MSC: mesenchymal stromal cell
mTOR: mammalian target of rapamycin
mTORC1: mammalian target of rapamycin complex 1
NF‐κB: nuclear factor‐κB
PAI1: plasminogen activator inhibitor 1
PI3K: phosphoinositide 3‐kinase
PKC: protein kinase C
ppWAT: periprostatic white adipose tissue
RAF: rapidly accelerated fibrosarcoma
RANKL: receptor activator of nuclear factor‐κΒ ligand
RTK: receptor tyrosine kinase
STAT: signal transducer and activator of transcription
TGFβ: transforming growth factor‐β
TNF: tumor necrosis factor
VAT: visceral adipose tissue
VEGF: vascular endothelial growth factor
WAT: white adipose tissue

## 1 Introduction

### 1.1 General Background

Prostate cancer (PCa) is the commonest cancer in men. In the U.S., after decades of declining incidence, PCa is increasing with alarmingly more cases of advanced disease. The American Cancer Society estimated nearly 300,000 PCa cases diagnosed in 2024, with over 35,000 deaths from this disease [1]. The impact of lifestyle is a key area of interest for reducing PCa incidence. Hence, obesity, conventionally defined by a body mass index (BMI) > 30 kg/m^2^ (> 25 kg/m^2^ in Asians) and addressed with lifestyle interventions, has garnered specific attention. Recently, a novel Adiposity‐Based Chronic Disease (ABCD) preventive care model has been developed based on abnormal adiposity amount, distribution (including ectopic fat), and function (e.g., adipokine secretion) along four sequential pathophysiological stages (1‐ risk [e.g., genetic/environmental/behavioral factors]; 2‐ predisease [e.g., overweight]; 3‐ disease [e.g., obesity]; and 4‐ complications [e.g., cardiovascular]) [2]. Various aspects of ABCD, including biological (e.g., metabolic syndrome) and nonbiological (e.g., social determinants of health [SDOH]) factors, have been identified as mechanistic/causal drivers of several cancers including PCa [3]. Additionally, a report from the World Cancer Research Fund summarized the mechanisms linking obesity and chronic inflammation with adipokines and signal transducer of activators of transcription (STAT) signaling causing genomic instability, angiogenesis, alterations in mitochondrial function, and insulin‐induced phosphatidylinositol‐3‐kinase/protein kinase B/mammalian target of rapamycin (PI3K/mTOR) and mitogen‐activated protein kinase*/extracellular signal‐regulated protein kinases* (MAPK/ERK) pathway activation facilitating cellular proliferation, resistance to cell death, and evasion of growth suppressors [3]. The molecular biology underlying the association between ABCD and PCa centers on chronic inflammation in both visceral white adipose tissue (WAT; VAT) and periprostatic WAT (ppWAT), with adipokines, chemokines, and cytokines promoting cellular growth pathways such as AMP‐activated protein kinase (AMPK) and mTOR [4]. Many of these molecular pathways are interpreted in isolation, but a more holistic, biological perspective, best presented as a network of relationships, may provide useful emergent information that can be hypothesis‐generating and eventually guide management decisions. To this end, we perform a preliminary review of epidemiological and mechanistic associations of ABCD (which includes but is not limited to obesity designations in the literature) and PCa to identify clinically relevant factors (i.e., adipokines and physiological events; nodes), and then present results of network and gene set enrichment analyses of predominant mechanistic drivers.

### 1.2 Epidemiology of Prostate Cancer and Abnormal Adiposity Relationships

The epidemiological relationships of obesity (ABCD stages 3 and 4) and PCa are complex [4]. Several studies have demonstrated a decreased incidence of low‐risk PCa in men with ABCD [5], associated with lower androgen levels, less aggressive screening in study populations, and high circulating volumes that reduce prostate‐specific antigen levels and thus the chance of prostate biopsy, as well as differences in varying ethnocultural populations [6]. Conversely, the Health Professions Follow‐Up Study examining nearly 2600 patients with localized PCa found that every pound of body weight gained per year in non‐smokers from age 21 years to diagnosis was associated with a higher chance of high‐risk PCa (HR 1.47, 95% CI 1.01–2.14) [7]. Moreover, a World Cancer Research Fund metanalysis (> 9 million men of whom 191,000 had PCa) demonstrated ABCD was associated with an 8%–11% higher risk of PCa metastatic disease and mortality [4]. Other studies, both retrospective and prospective, have shown a positive association between increased biochemical recurrence risk and BMI after either surgery or external beam radiotherapy for localized PCa [8].

### 1.3 Adiposity‐Based Chronic Disease

Adipose tissue is one of the body's largest endocrine organs, accounting for, on average, 38% of female and 26% of male body weight [9]. Excess adipose tissue is defined by BMI and then designated as overweight or obesity, but this excludes other abnormal adiposity descriptors based on body distribution and adipocyte function. In contrast to the overweight/obesity designations, the ABCD model is less stigmatizing, more expansive, and codifies statistical risk factors, mechanistic drivers, SDOH, and staged pathophysiological progression to more explicitly direct preventive care measures [2]. Both WAT and brown adipose tissue exist, the former being more widespread and implicated in oncogenesis, while the latter, characterized by increased cytoplasmic mitochondria and lipid droplets, is believed to be involved in normal body energy metabolism [10]. Further differentiation of VAT from subcutaneous fat is important: VAT has a higher risk of fibrosis with ABCD‐related inflammation and higher expression of genes related to adipogenesis and insulin pathways [11]. Also, VAT encompasses ectopic adipose tissue, defined by anatomical location and exemplified by ppWAT (a layer of adipose tissue surrounding approximately half of the prostatic capsule and present in higher volumes in patients with ABCD) [6, 12]. The clinical significance of ppWAT and its secretome stems from anatomical proximity and crossing vascularity with developing PCa cells constituting a tumor microenvironment (TME) [13]. In fact, prospective clinical studies of patients with newly diagnosed PCa found nearly 50% had ppWAT inflammation, which was significantly associated with higher BMI and Gleason grade scores [14].

### 1.4 Mechanistic Relationships Between Prostate Cancer and Abnormal Adiposity

Chronic inflammation in ABCD is a principal driver of PCa risk. The cellular makeup of VAT is 90% adipocytes, with other cells such as adipocyte stromal cells (ASCs), immune cells (leukocytes and macrophages), and endothelial cells forming the rest in the stromal vascular fraction [15]. As WAT expands with ABCD, adipocytes outgrow their blood supply, become hypoxic, facilitate hypoxia inducible factor 1α (HIF1α) activation, and induce angiogenesis [16]. Local ischemia triggers macrophage proliferation and coalescence, resulting in crown‐like structures around dying adipose cells and recruitment of other leucocytes [17], as well as a shift in macrophage phenotype from anti‐inflammatory M2 to pro‐inflammatory M1 cell types [18]. This cascade of events results in increased secretion of bioactive proinflammatory molecules, including cytokines (tumor necrosis factor‐α [TNFα] and interleukin [IL]‐6, IL‐8), chemokines (C‐C motif chemokine ligand 7 [CCL7], chemokine [C‐X‐C motif] ligand 1 [CXCL1], CXCL12, and monocyte chemoattractant protein‐1 [MCP‐1]), growth factors (leptin, adiponectin, vascular endothelial growth factor [VEGF], hepatocyte growth factor [HGF], transforming growth factor beta [TGFβ], and fibroblast growth factor [FGF]), and other bioactive compounds such as plasminogen activator inhibitor 1 (PAI1), facilitating both chronic inflammation, and PCa oncogenesis/progression [6]. Chronic inflammation in ABCD can trigger insulin resistance, although some studies suggest this relationship is bidirectional [19]. ABCD‐associated insulin resistance can promote insulin‐like growth factor (IGF), leptin, and adiponectin signaling, which may increase PCa risk [20], and TNFα, IL‐1β, and IFNδ from T‐cells and macrophages act through cell surface receptors to stimulate intracellular pathways that promote insulin resistance [19]. Also, in mouse models, obesity‐associated IGF‐1 is believed to promote PCa oncogenesis via PI3/AKT, Forkhead Box 0 3A (FOXO3A), and B‐*cell* Lymphoma 2‐like protein 11 (BIM) signaling [21], and both in vitro and in vivo studies of IGF‐1 receptor activation in PCa reveal angiogenesis and activation of cellular survival pathways via a5b1 integrin expression [22].

## 2 Methods

### 2.1 Preliminary Review

**FIGURE 1:**
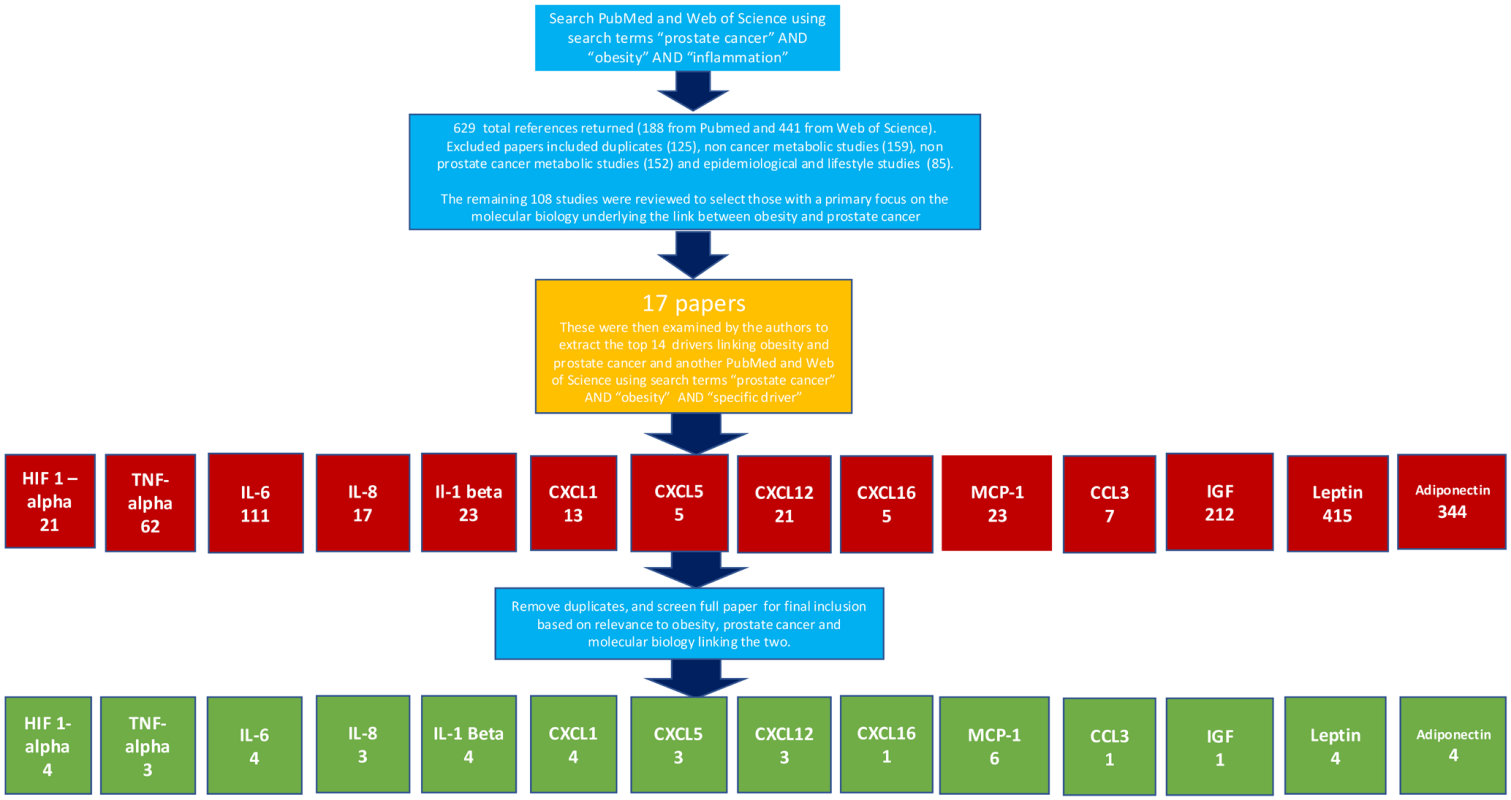
Diagram of Preliminary Review Methodology used to identify the 14 most prominent biomarkers in the ABCD‐PCa network.

Web of Science and Pubmed databases were initially queried using “prostate cancer” AND “obesity” AND “inflammation” as search terms. Obesity was chosen as the best search term for ABCD based on the popularity and preference of this term in studies conducted on adiposity and cancer. This search yielded a total of 629 references (188 from Pubmed and 441 from Web of Science). Following the initial search, references were excluded if they were duplicate studies (125), studies focused on metabolism and inflammation without a focus on cancer (159), studies focused on cancers other than prostate (152), or epidemiological studies and clinical studies relating to lifestyle [23]. The remaining 108 references were reviewed to select those with a primary focus on the molecular biology underlying the link between obesity and PCa, as well as those providing explanatory details of dominant metabolic and inflammatory drivers. Applying these criteria, 17 reviews were selected for detailed examination, and the mechanistic drivers outlined were cataloged. A second query was then performed using Web of Science and PubMed databases with “prostate cancer” AND “obesity” AND “driver” as search terms. Once again, these references were reviewed by the authors to select those with a primary focus on the role of specific metabolic drivers in the link between obesity and PCa, specifically outlining their cellular origin, signaling pathway, and oncogenic effect. After duplicate references were removed and secondary references (cited in the primary search papers, which may have included research on cancers other than PCa) added, the following predominant drivers (by descending number of respective references) were identified: MCP‐1 (6), CXCL1 (4), IL‐1β (4), adiponectin (4), leptin (4), HIF1α (4), IL‐6 (4), IL‐8 (3), TNFα (3), CXCL12 (3), CXCL5 (3), CCL3 (1), and IGF‐1 (1). CXCL16 was also considered based on its prevalence in the literature with respect to PCa and inflammation but without specific reference to ABCD. This methodology is illustrated in more detail in the flow chart in Figure 1.

### 2.2 Network Analysis

The network was constructed by defining relationships of the predominant drivers found in the preliminary review. The bioinformatic online tool Web‐based Gene SeT AnaLysis Toolkit (WebGestalt [24]) was used to generate a Network Topology‐based Analysis using 494 public PCa tissue RNA sequencing (RNAseq) gene expression data from The Cancer Genome Atlas (TCGA). Briefly, webgestalt.org was accessed, the method “Network Topology‐based Analysis” was selected together with the “Network” functional database and “TCGA RNASeq PRAD.” The list of biomarkers was entered into the designated box for gene lists on the website. “Network Retrieval & Prioritization” was selected with default advance parameters. This method maps how genes connect with each other based on co‐expression to uncover the most linked genes as potential key players. Results were downloaded for their use in the work. The analysis generated information about the biomarker interactions based on PCa tissue data and the biomolecular interactions based on Gene Ontology (GO) categories. The network topology metrics used are betweenness centrality and modularity, which are both seen in the results figures. Biomarker connections in the network are based on co‐expression of their associated genes, with additional functional hierarchical co‐expression represented by the betweenness centrality score. The higher the betweenness centrality score, the greater the number of co‐expression connections a biomarker has within the network, with the implication being it is the most influential. Betweenness centrality measures how connected a biomarker is based on how often it appears in the shortest path to other biomarkers, and is calculated by identifying all the shortest paths, counting how many of the shortest paths passed through a biomarker, and dividing this number by the total number of shortest paths in the network using the function *betweenness_centrality* from the *NetworkX* library in Python. Modularity measures the strength of division of the network: modularity of 1 means the network is divisible into perfect communities with no links between them, while 0 means that network division into communities is equivalent to random grouping. Modularity was calculated using the algorithm *modularity*, part of the *NetworkX* library in Python. The code for these calculations can be found in GitHub (https://github.com/etomasbort/ABCD‐Prostate‐Cancer‐Oncogenic‐Network.git). To assess robustness, we repeated the analysis using FunMap, an independent cancer omics database, and confirmed that the top key players remained consistent across datasets [25]. Subnetworks were generated by identifying the desired adipokine biomarkers and their respective connections to create a “zoom in” from the main ABCD‐prostate cancer oncogenic network. Microsoft PowerPoint was used to design and assemble the network analysis results and respective subnetwork figures.

Gene Set Enrichment Analysis (GSEA) was used to predict the molecular functions of the main network biomarkers. PCa RNAseq data from TCGA was accessed using the online cancer genomic database cBioPortal [26] and a differential expression analysis was performed using the “Explore Selected Studies” function, comparing two patient groups split by the median expression of the desired biomarker (high vs. low expression). Differential expression analysis determined the changes in expression levels between the groups being compared. The differential expression analysis results were copied and pasted into WebGestalt at webgestalt.org, with the GSEA method selected and “Geneontology,” “Molecular Function noRedundant” chosen as “Functional Database.” The GSEA analysis then revealed the top molecular functions enriched in PCa with high expression of the desired biomarker.

## 3 Results

### 3.1 Preliminary Review

The current state of knowledge on the molecular biology linking ABCD and PCa suggests excess adiposity results in hypoxia and increased HIF‐1α signaling. Insufficient angiogenesis causes adipocyte cell death, activation of proinflammatory MI macrophages, and release of pro‐inflammatory cytokines, chemokines, and adipokines. Alongside this process, lipids accumulate in/around vital organs (e.g., VAT) and periprostatic tissues (e.g., ppWAT) contributing to insulin resistance and metabolic syndrome. The availability of lipids provides a source of fatty acids for membranes and bioactive compounds, supplies an alternate source of energy (other than glucose) for PCa cells, and participates in the TME. Within this milieu, 14 key drivers were identified, defined by cancer hallmarks such as the cell of origin, signaling pathway, oncogenic effect, and specific effect (Table 1). These drivers are produced by a broad range of cell types, including adipocytes, ASCs, immune cells including macrophages, endothelial cells, epithelial cells, fibroblasts, and cancer cells themselves. The oncogenic effects of specific signaling pathways instigated by these drivers include tumor initiation, tumor progression, matrix remodeling, epithelial‐mesenchymal transition (EMT), and metastasis which can be related to cellular proliferation (HIF1α, IL‐6, CXCL5, CXCL12, CXCL16, MCP‐1, CCL3, and IGF), resisting cell death (HIF1α, IL‐6, CXCL12, IGF‐1, and leptin), metabolic reprogramming (HIF1α), tumor‐promoting inflammation (TNFα, CCL3, and leptin), avoiding immune destruction (TNFα, IL‐6, and leptin), angiogenesis (HIF1α, IL‐6, CXCL1, IGF‐1, and leptin), and activating invasion (TNFα, IL‐6, IL‐1β, CXCL1, CXCL5, CXCL12, CXCL16, MCP‐1, IGF‐1, and leptin) [75]. By contrast, adiponectin may protect against activating invasion and sustained proliferative signaling.

**TABLE 1:** Biomarkers and cancer hallmarks in the ABCD‐prostate cancer oncogenic network.^a^

Biomarkers	Cells of origin [6]	Mechanistic pathways	Oncogenic effect	Effect as cancer hallmark	References
HIF1α	Adipocytes, innate and adaptive immune cells (macrophages, neutrophils, dendritic cells, and lymphocytes)	Phosphatidylinositol‐3‐kinase/protein kinase B/Mammalian target of rapamycin (PI3K/Akt/mTOR), Nicotinamide adenine dinucleotide phosphate (NADPH) oxidase (NOX), Wingless‐type MMTV integration site family (WNT)/Beta‐catenin and Hedgehog signaling	Mediate hypoxia driven metabolic reprogramming of adipocytes and the tumor microenvironment (TME) to fuel cancer cell proliferation, angiogenesis, antiapoptosis, cell survival and epithelial‐mesenchymal transition (EMT)	Sustained proliferative signaling Inducing vasculature Resisting cell death Metabolic reprogramming	[27, 28, 29, 30]
TNFα	Macrophages, adipose stromal cells (ASCs), Natural Killer (NK) Cells, CD4 T helper cells, as well as developing cancer cells within the TME	Nuclear factor‐kB (NK‐kB), mitogen‐activated protein kinase (MAPK) pathway, apoptosis, and phosphatidylinositol‐3‐kinase/protein kinase B (PI13/Akt) for epithelial‐mesenchymal transition (EMT)	Chronic inflammation, insulin resistance, CD8+ T cell death, and EMT to promote invasion and metastatic progression	Avoiding Immune destruction Tumor promoting inflammation Activating invasion and metastasis	[31, 32, 33]
IL‐6	Macrophages, monocytes, T Cells, endothelial cells, other activated immune cells, stromal cells (including ASCs) and fibroblasts	Janus kinase (JAK)‐signal transducer of activators of transcription (STAT) (JAK/STAT), MAPK and PI3K/Akt pathways	Cellular proliferation, cell survival, angiogenesis, invasiveness and metastasis and immunosuppression	Sustaining proliferative signaling Avoiding immune destruction Activating invasion and metastasis Inducing vasculature Resisting cell death	[34, 35, 36, 37]
IL‐8 (CXCL8)	Adipocytes, ASCs, macrophages, endothelial, epithelial, mesothelial, fibroblasts and tumor cells	NF‐kB, JAK/STAT3 and MAPK pathways	EMT, TAMs infiltration, local invasion, and metastasis	Tumor promoting inflammation Activating invasion and metastasis	[38, 39, 40]
IL‐1β	Fibroblasts, immune and cancer cells	NF‐KB, endothelin‐1 and (ET‐1) signaling with reduced androgen receptor expression	Disease progression, metastasis, and treatment resistance	Activating invasion and metastasis	[41, 42, 43, 44]
CXCL1	ASCs, neutrophils, macrophages, and tumor cells	Extracellular signal‐regulated protein kinases 1 and 2 (ERK1/2), Epidermal growth factor (EGF), Nuclear factor kappa B/sex determining region Y‐box 4 (NF‐kB/SOX4), Proto‐oncogene tyrosine‐protein kinase Src (SRC)	Angiogenesis, EMT, tumor cell migration and invasion	Inducing vasculature. Activating invasion and metastasis	[45, 46, 47, 48]
CXCL5	ASCs, monocytes, macrophages, neutrophils, epithelial, fibroblastic and endothelial cells	Rapidly accelerating Fibrosarcoma (Raf)/mitogen activated protein kinase (MEK)/ERK pathway, Early Growth Response 1 (EGR‐1), SNAIL pathways	Cellular proliferation, EMT and invasion	Sustaining proliferative signaling Activating invasion and metastasis	[49, 50, 51]
CXCL12	Endothelial cells, adipocytes, ASCs and tumor cells	PI3K/AKT, Src, JNK and ERK pathways	Cellular proliferation, metastatic behavior and survival	Sustaining proliferative signaling Activating invasion and metastasis Resisting cell death	[52, 53, 54]
CXCL16	Adipocytes, tumor cells and dendritic cells	PI3/Akt, ERK and MAPK pathways	Cellular proliferation, migration, EMT	Sustaining proliferative signaling Activating invasion and metastasis	[55, 56]
Monocyte Chemoattractant Protein‐1 (MCP‐1)	Fibroblasts, endothelial, epithelial, smooth muscle and mesangial cells, adipose cells, and ASCs	PI3/Akt, Mammalian target of rapamycin complex 1 (MTORC1) and Rho GTPase‐activating protein (Rac) pathways	Cellular proliferation, migration, disease progression and cabazitaxel resistance	Sustaining proliferative signaling Activating invasion and metastasis	[57, 58, 59, 60]
CCL3 (MCP‐1 alpha)	Epithelial cells, fibroblasts, monocytes, macrophages, lymphocytes, and WAT cells	ERK and Akt pathways	Oncogenesis via inflammation promoting cellular proliferation	Sustaining proliferative signaling Tumor promoting inflammation	[61]
IGF‐1	Hepatocytes	Pi3/Akt/Forkhead Box 0 3A (FOXO3A)/B‐cell Lymphoma 2‐like protein 11 (BIM) signaling	Angiogenesis, cell survival growth and invasion	Sustaining proliferative signaling Inducing vasculature Resisting cell death Activating invasion and metastasis	[22]
Leptin	Adipocytes and ASCs	MAPK, STAT and PI3K/MTOR pathways	Inflammation, blocking apoptosis, angiogenesis and immune suppression	Tumor promoting inflammation Resisting cell death Inducing vasculature Avoiding Immune destruction	[62, 63, 64, 65]
Adiponectin	Adipocytes (VAT)	Peroxisome proliferator‐activated receptor (PPAR) and AMP‐activated protein kinase (AMPK) that inhibit mTOR, PI3K and STAT3 (the later via suppressing cytokine signaling 3 (SOCS3)) (Bocian)	Protective by inhibition cancer cell proliferation and progression	Protective against Sustained proliferative signaling Activating invasion and metastasis	[62, 63, 64, 65]

^a^ Cancer hallmarks comprise the functional capabilities of cancer cells. Information compiled from selected reviews [3, 4, 6, 10, 19, 20, 23, 66, 67, 68, 69, 70, 71, 72, 73, 74].

### 3.2 Modular Adiposity—Prostate Cancer Network Analysis and GSEA

**FIGURE 2:**
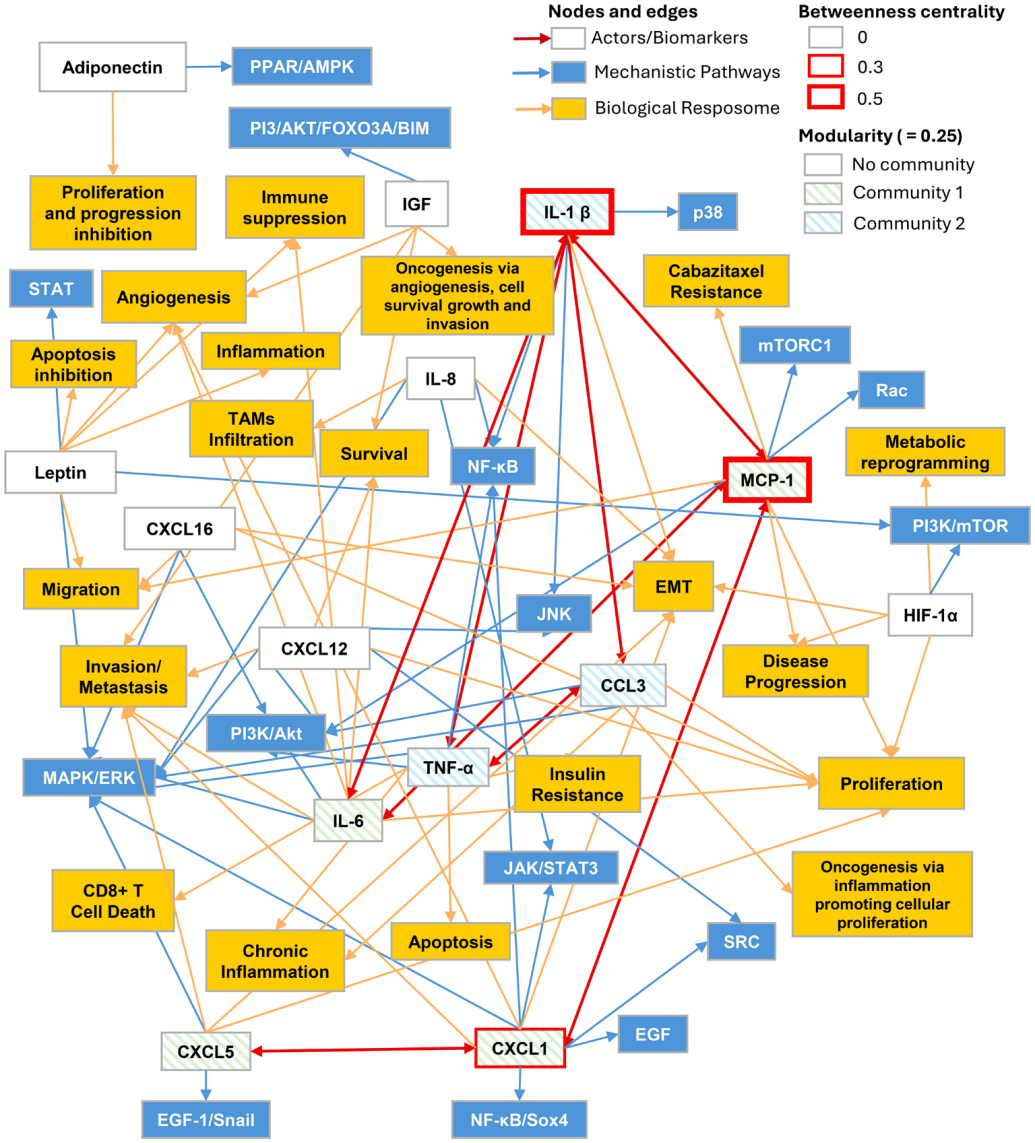
The ABCD‐Prostate Cancer Network. The findings of the Network Analysis suggest the most influential biomarkers, based on highest connectivity, are MCP‐1, IL‐1β, and CXCL‐1. Also, two dominant network communities emerge: The MCP‐IL‐6‐CXCL1‐CXCL5 and IL‐1β‐TNFα‐CCL3 signaling pathways. These highly connected biomarkers and their two dominant subcommunities induce biological effects that promote PCa development and progression. The network was constructed from Table 1 defining connections between biomarkers, mechanistic pathways and biological effects. The biomarkers are in white boxes and red arrows indicate co‐expression which was found in the network topology‐based analysis. Mechanistic pathways are in blue boxes and biological effects are in yellow boxes. These connections are derived from the literature review findings that are presented for each biomarker in Table 1. As defined in the diagram Nodes are connection points or entities and Edges are links between nodes that convey information. Betweenness centrality measures how connected a biomarker is in the network based on how often it appears in the shortest path to the other biomarkers (nodes), and was calculated by identifying all shortest paths, counting how many of the shortest paths passed through a node, and dividing this number by the total number of shortest paths in the network using the function *betweenness_centrality* from the *NetworkX* library in python. Modularity measures the strength of division of the network: Modularity of 1 means the network is divisible into perfect communities with no links between them, while 0 means that network division into communities is equivalent to random grouping. Modularity was calculated using the algorithm *modularity*, part of the *NetworkX* library in python. The communities identified by the algorithm are shown by coloring the background of nodes with differently colored stripes. Uncolored nodes correspond to categories with a single biomarker.

The results of the Modular Network Analysis suggest the most influential biomarkers, based on highest co‐expression connectivity, are MCP‐1, IL‐1β, and CXCL‐1 (Figure 2). Interestingly, two dominant network communities emerge: the MCP‐IL‐6‐CXCL1‐CXCL5 and IL‐1β‐TNFα‐CCL3 signaling pathways. The top GO categories for the overall network (molecular function, biological process, and cellular component) demonstrate two main functions—inflammation (cytokine‐mediated signaling, leukocyte migration, leukocyte chemotaxis, myeloid leukocyte migration, and positive regulation of neuroinflammatory response) and immune response to bacteria (cellular response to biotic stimulus, cellular response to molecule of bacterial origin, cellular response to lipopolysaccharide [LPS], humoral immune response, and neutrophil chemotaxis) (Table 2). The Top 5 GO molecular functions for the most influential biomarkers in the ABCD‐prostate cancer oncogenic network (IL‐1β, MCP‐1, and CXCL1) are consistently related to glycosaminoglycan/integrin binding and immune receptor activity (predominantly as IL‐1β function) (Table 3). Both IL‐1β and MCP‐1 demonstrate an extracellular matrix structural constituent function, and MCP‐1 and CXCL1 show antigen binding function. Broadly speaking, these functions may relate to inflammation, the immune response to bacteria, and oncogenesis with development of the TME, including cellular invasion, angiogenesis, cellular proliferation, and metastatic progression. Although the molecular functions of IL‐1β, MCP‐1, and CXCL1 revealed by the GSEA may not be exactly the same as the oncogenic effects suggested by the preliminary review (e.g., MCP‐1 for cellular proliferation and tumor invasion; IL‐1β for EMT and tumor invasion; and CXCL1 for angiogenesis, EMT, and tumor invasion), they are not unrelated with respect to cell invasion, angiogenesis, cellular proliferation, and metastatic progression.

**TABLE 2:** Top 10 Gene Ontology (GO) categories for the ABCD‐prostate cancer oncogenic network.^a^

GO ID^b^	GO name^c^	Overlapping genes from list/Genes in category^d^	FDR^e^
GO:0050900	Leukocyte migration	7/241	< 0.0001
GO:0006959	Humoral immune response	6/111	< 0.0001
GO:0150078	Positive regulation of neuroinflammatory response	4/11	< 0.0001
GO:0019221	Cytokine‐mediated signaling pathway	7/287	< 0.0001
GO:0071222	Cellular response to lipopolysaccharide	6/123	< 0.0001
GO:0071219	Cellular response to molecule of bacterial origin	6/131	< 0.0001
GO:0097529	Myeloid leukocyte migration	6/143	< 0.0001
GO:0030595	Leukocyte chemotaxis	6/147	< 0.0001
GO:0071216	Cellular response to biotic stimulus	6/149	< 0.0001
GO:0030593	Neutrophil chemotaxis	5/62	< 0.0001

^a^ See Figure 2 for network depiction.

^b^ GO ID is the unique identifier associated with a GO category, which is a list of genes associated with a molecular function, biological process, or cellular component.

^c^ GO Name is the name assigned to a GO category, corresponding to a molecular function, biological process, or cellular component.

^d^ Overlapping genes from list/Genes in category is the number of genes in the ABCD‐prostate cancer oncogenic network that overlap with the genes found in the different GO categories.

^e^ False discovery rate (FDR) is determined by the *p* value. With network and gene category analysis, where thousands of comparisons are performed, the potential for false discovery is high. To mitigate this risk, the *p* value is corrected with the Benjamini and Hochberg method to create a *p* value adjusted to correct for multiple comparisons. FDR < 0.05 is statistically significant.

**TABLE 3:** Top 5 GO molecular functions for the most connected biomarkers in the ABCD‐prostate cancer oncogenic network.^a^ ^,^ ^b^

Actor/Biomarker^c^	GO ID^d^	GO name^e^	Leading edges IDs/Gene set size^f^	Normalized enrichment score^g^	FDR^h^
IL‐1β	GO:0140375	Immune receptor activity	103/140	1.800	< 0.0001
	GO:0005178	Integrin binding	97/157	1.720	0.0003
	GO:0005201	Extracellular matrix structural constituent	106/161	1.647	0.0005
	GO:0005539	Glycosaminoglycan binding	132/234	1.646	0.0005
	GO:0030546	Signaling receptor activity	234/486	1.551	0.0052
MCP‐1	GO:0140375	Immune receptor activity	93/140	1.688	< 0.0001
	GO:0003823	Antigen binding	48/65	1.627	0.0005
	GO:0005201	Extracellular matrix structural constituent	116/162	1.600	0.0012
	GO:0005178	Integrin binding	97/157	1.584	0.0015
	GO:0005539	Glycosaminoglycan binding	132/234	1.568	0.0030
CXCL1	GO:0140375	Immune receptor activity	99/140	1.654	0.0047
	GO:0003823	Antigen binding	42/65	1.616	0.0047
	GO:0061134	Peptidase regulator activity	90/215	1.505	0.0185
	GO:0005178	Integrin binding	96/157	1.486	0.0237
	GO:0015026	Glycosaminoglycan binding	138/234	1.461	0.0370

^a^ See Figure 2 for network depiction.

^b^ Explanation of differences between Tables 2 and 3. Table 2 considers all biomarkers as a whole, whereas Table 3 considers the most connected biomarkers individually. While Table 2 answers “What molecular functions does this network affect as a whole” Table 3 answers “what do the most influential biomarkers in this network (IL‐1β, MCP‐1, and CXCL1) do?” For the overall network, the functions are mostly associated with inflammation and the immune response to LPS and bacteria, which hints at a possible role of diet and gut and urinary microbiota in the link between ABCD and prostate cancer. Examining Table 3, presenting the most influential biomarkers, their molecular functions relate to inflammation but also invasion, for example, integrin binding, extracellular matrix structural constituent, and glycosaminoglycan binding, hinting at a possible role in disease progression and metastasis.

^c^ Actor/biomarker is the biomarker from the ABCD‐prostate cancer oncogenic network analyzed via a gene set enrichment analysis (GSEA) based on high or low biomarker expression in prostate cancer tissues (*n* = 494) from The Cancer Genome Atlas (TCGA).

^d^ GO ID is the GO unique identifier for a list of genes or gene sets related to a molecular function.

^e^ GO name is the GO molecular function gene set name.

^f^ Leading edge IDs is the number of genes contributing to the enrichment score.

^g^ Normalized Enrichment Score indicates how overrepresented a molecular function is in prostate cancer tissues with high expression of the actor/biomarker compared to randomized gene lists.

^h^ False discovery rate (FDR) is a probability test value adjusted for multiple comparisons using the Benjamini‐Hochberg method. This adjustment helps identify false positives, ensuring that the significant results are more reliable. FDR < 0.05 is statistically significant.

### 3.3 Adipokine Subnetwork

**FIGURE 3:**
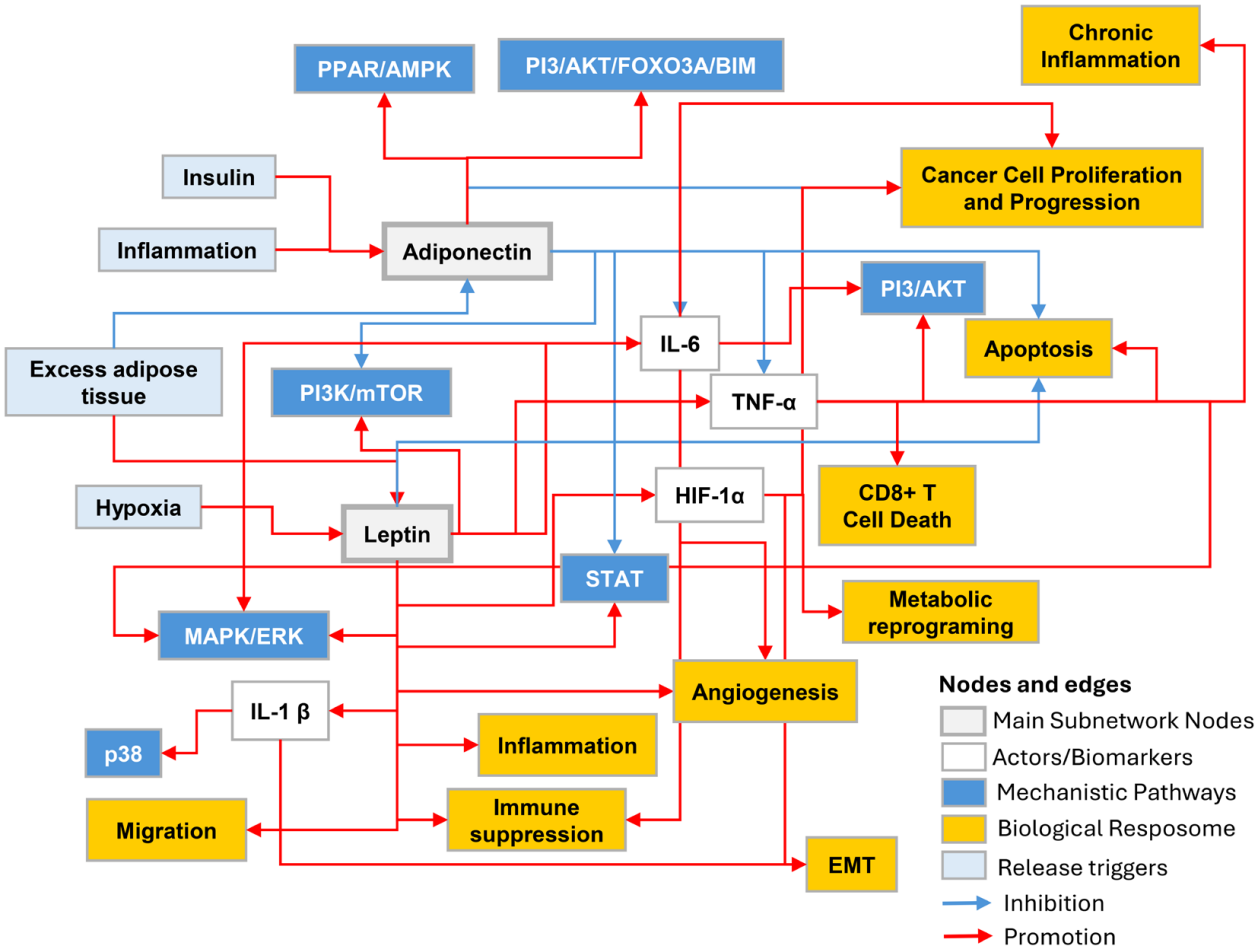
Leptin and adiponectin ABCD‐prostate cancer subnetwork. These adipokines exert opposing relationships on ABCD and PCa with higher levels of adiponectin decreasing PCa risk and higher levels of leptin increasing PCa risk. Leptin and adiponectin are the main nodes of this subnetwork, depicted in gray boxes. Adiponectin activates cellular functions and pathways via peroxisome proliferator‐activated receptor (PPAR)/AMPK that inhibit mTOR, PI3K, and STAT3 inhibiting cancer cell proliferation and progression. Conversely, leptin promotes pathways and cellular functions contributing to PCa risk with activation of MAPK, STAT, and PI3K/MTOR, resulting in inflammation, blocking apoptosis, angiogenesis, and immune suppression. It is notable that the network analysis found adiponectin and leptin were not highly connected within the ABCD‐PCa network. Adiponectin only has an inhibitory connection to TNF alpha, and Leptin has activating connections to HIF‐1 alpha, TNF alpha, IL‐6 and IL‐1 Beta. Neither is connected to MCP‐1 or CXCL‐1 and they do not appear in the 2 dominant sub communities illustrated in Figure 2. Leptin and adiponectin connections to other biomarkers in white boxes are derived from the network topology‐based analysis from Figure 2. The connections to mechanistic pathways in blue boxes and the biological effects in yellow boxes are derived from the literature search presented in Table 1. The arrows indicate inhibitory (blue) or activating (red) relationships with the other nodes are also derived from the literature search presented in Table 1. Light blue boxes represent phenomena related to the release of adiponectin or leptin.

Analysis of the leptin and adiponectin ABCD‐PCa subnetwork shows that these adipokines exert opposing relationships on ABCD and PCa, with higher levels of adiponectin decreasing PCa risk and higher levels of leptin increasing PCa risk (Figure 3). Adiponectin activates cellular functions and pathways via peroxisome proliferator‐activated receptor and AMPK that inhibit mTOR, PI3K, and STAT3 (the last via suppressing cytokine signaling 3), inhibiting cancer cell proliferation and progression. Conversely, leptin promotes pathways and cellular functions contributing to PCa risk with activation of MAPK, STAT, and PI3K/MTOR, resulting in inflammation, blocking apoptosis, angiogenesis, and immune suppression either alone or synergistically with VEGF. However, based on what is presented in the overall network analysis, the strength of leptin and adiponectin as biomarkers connecting ABCD and PCa risk is less significant than that of IL‐1β, MCP‐1, and CXCL1.

## 4 Discussion

A preliminary review of the literature supports the role of chronic inflammation in the link between ABCD and PCa risk, triggered by hypoxic adipocytes with HIF‐1α signaling, adipocyte cell death, and activation of M1 (proinflammatory) macrophages in VAT and ppWAT. Furthermore, current research has demonstrated that, alongside the development of chronic inflammation, adipokines, chemokines, and cytokines secreted by adipocytes, ASCs, immune cells (including macrophages), and cancer cells create a gradient of bioactive compounds among VAT, ppWAT, and the prostate. These drivers activate signaling pathways such as MAPK, PI3K/Akt/mTOR, STAT, and NF‐kB to induce cellular proliferation, anti‐apoptosis, metabolic reprogramming, EMT, tumor‐promoting inflammation, immune destruction, angiogenesis, and tumor invasion [75]. Our modular network analysis of the 14 most prominent drivers in this process found that the most connected biomarkers were IL‐1β, MCP‐1, and CXCL1, with two dominant communities within the overall network centered around MCP, IL‐6, CXCL1, and CXCL5 signaling and IL‐1β, TNFα, and CCL3 signaling. Biomarker connections in the network (Figure 2) are based on co‐expression of their associated genes, with additional functional hierarchical co‐expression represented by the betweenness centrality score. The higher the betweenness centrality score, the greater the number of co‐expression connections a biomarker has within the network, with the implication being it is the most influential. At the core of one sub community, MCP‐1 has strong connectivity with IL‐6 and CXCL‐1, which is itself connected to CXCL‐5, and from the preliminary review, we can hypothesize this facilitates mTORC1 and Rac (MCP‐1), EGF, SRC, NF‐kB/Sox4, and JAK/STAT3 (CXCL‐1), MAPK/ERK and EGF‐1/Snail (CXCL‐5), and MAPK/ERK and PI3/Akt (IL‐6) signaling. The downstream effects of the subnetwork influence such cancer hallmarks and biological effects as immune suppression, cell survival, cellular proliferation and migration, angiogenesis, EMT, invasion, and disease progression and via MCP‐1 potentially cabazitaxel resistance. In the other subnetwork, IL‐1 Beta connects to TNF‐alpha and CCL3 (who also connect to each other), which again, from the preliminary review, we can hypothesize influences JNK, p38, NF‐kB (IL‐1 Beta), MAPK/ERK, NF‐kB, and PI3/Akt (TNF alpha) and MAPK/ERK and PI3/Akt (CCL3) signaling. The downstream effects of the second subnetwork include EMT, insulin resistance, inflammation, apoptosis, activation of CD8+ T cells, chronic inflammation, and cellular proliferation. Moreover, the two subnetworks are connected directly between MCP‐1 and IL‐1 Beta and through IL‐6. GSEA to uncover overlapping gene sets within GO categories again supports the role of chronic inflammation of the overall network demonstrating molecular functions relating to chronic inflammation but also an immune response to bacteria and LPSs. For the most connected biomarkers (IL‐1β, MCP‐1, and CXCL1), GSEA found GO categories with molecular functions such as glycosaminoglycan and integrin binding, immune receptor activity, and extracellular matrix functional constituents (as a part of ECM remodeling), which may all relate to inflammation, the immune response to bacteria, and PCa progression within the TME. A recent review suggested recruitment of ASCs from ppWAT into PCa cells and the TME are precursors to tumor stromal cells and cancer‐associated fibroblasts and a source of IL‐6, TNFα, CXCL1, CXCL5, CXCL8, CXCL12, MCP‐1, and leptin facilitating tumor angiogenesis and cancer cell proliferation [6]. CXCL1 has been shown to be released by human and mouse PCa cells and, acting via its CXCR1 receptor, facilitates recruitment of ASCs into prostate tumors [76]. Moreover, ASC recruitment to PCa tumor cells and the TME may be brought about by the actions of CXCL1 and MCP produced by the cancer cells themselves [6].

Based on the results of the modular network analysis, adiponectin, leptin, and IGF1 signaling have less significant roles in the overall network. The adiponectin and leptin subnetworks demonstrate opposing roles in ABCD and PCa; however, in the overall network, adiponectin, leptin, and IGF1 are not highly connected and do not feature within the two prominent communities. Leptin, secreted from adipocytes and ASCs, has been extensively studied, and via MAPK, STAT, and PI3/MTOR pathways, promotes inflammation, anti‐apoptosis, angiogenesis, and immune suppression [62] (Table 1). In its subnetwork, leptin is linked to HIF‐1α, IL‐1β, and IL‐6, suggesting some influence over the control of those drivers' downstream effects such as EMT and tumor invasion (Figure 3). Insulin resistance and IGF1 are also thought to play an important role in ABCD‐linked PCa risk via PI3/AKT, FOXO3A, and BIM signaling, causing angiogenesis, cell survival, growth, and invasion [22]. However, IGF1 is primarily produced by hepatocytes, and it may be that human systemic endocrine signaling is less influential on PCa risk than in vitro and animal model studies on IGF1 would suggest. Alternatively, IGF1 signaling pathways influencing PCa risk may operate outside of the specific ABCD‐PCa network that has been studied.

In the hierarchy based on reduced risk of false discovery, the top GO categories emphasize molecular functions of the highlighted drivers in the ABCD‐PCa network relating to leucocyte migration, the immune response, and cellular responses to LPSs and molecules of bacterial origin (Table 2). This is an interesting finding and suggests an important additional source of systemic and local inflammation other than excess adiposity. Gut microbiome dysbiosis in ABCD as well as ppWAT and the prostate microbiome may result in exposure to bacteria and LPSs. Recent reviews have outlined numerous epidemiological investigations finding an association between increased PCa risk and gut and urinary microbiota [77, 78] and microbiota‐induced genetic toxins causing transmembrane serine protease 2—erythroblastosis virus E26 related gene fusions, which is known to be an early genetic event in PCa oncogenesis [78]. Firmicutes and Bacteroidetes are the commonest gut bacteria, as well as Verrucomicrobia, Fusobacteria, Proteobacteria, and Actinobacteria [79]. Another review explored the impact of gut dysbiosis on the immune system and its influence on PCa risk as well as actionable manipulation of gut microbiota with dietary phytochemicals to limit PCa risk and improve emotional and physical health [80]. Gut dysbiosis may cause a “leaky gut” due to altered tight junction protein expression (zonula occludens‐1 [ZO‐1] and occludin) with systemic leakage of bacterial components such as LPS and gut metabolites for example, SCFAs facilitating inflammatory signaling through activation of Toll Like Receptor 4 (TLR4) [77, 80]. With regard to the prostate microbiome, DNA sequencing has identified urinary bacteria such as *Corynebacterium*, *Anaerococcus*, *Streptococcus*, *Staphylococcus*, *Lactobacillus*, and *Propionibacterium*, as well as fungi, viruses, and protozoa [81] The prostatic epithelium can also be injured, exposing these organisms to prostatic tissue, again potentially activating Toll Like Receptor 4 (TLR4) on immune cells and exacerbating inflammatory signaling from the ppWAT [82] LPS in a high‐fat diet may also increase leptin [83], activating inflammatory cytokines such as TNFα, IL‐1β, and IL‐6 [84]. Moreover, leptin's role in inflammation is bidirectional. Both infectious and inflammatory stimuli, such as LPS, TNF‐α, and IL‐1β can elevate leptin levels, which may perpetuate the loop of chronic inflammation [85, 86], and it may be that leptin signaling can influence PCa risk via a network distinct from the ABCD—PCa network, relating to dietary exposure to LPS and the gut or prostate microbiome. Furthermore, improvements in diet, exercising to reduce body fat, and a reduction in both bacterial and LPS exposure to limit inflammation may all help to mitigate PCa risk relating to ABCD‐gut microbiome dysbiosis and the prostate microbiome. There have been several studies focused on diet as well as exercise to reduce excess adiposity and their influence not only on PCa tumor biology but also oncological outcomes. In the biomarker space, studies have examined inflammatory markers such as neutrophil to lymphocyte and platelet to lymphocyte ratios as well as TNF‐alpha and IL‐6, although results demonstrating their predictive power have been inconclusive [87]. American Cancer Society guidelines recommend vegetables, fruits, and whole grains while avoiding red and processed meats, added sugar, and refined grain products to maintain a healthy gut microbiome [88] and lifestyle programs incorporating moderate to vigorous physical activity do seem to improve oncological outcomes [87]. This is supported by clinical studies such as the PREDIMED trial examining the benefits of a Mediterranean diet in breast cancer patients [89] or the Ornish Study (a lifestyle program including a plant‐based diet and exercise regime) in PCa patients [90]. More research is required to assess the effects of specific dietary modifications or exercise programs on markers of systemic inflammation or how these kinds of programs can affect gut dysbiosis, systemic LPS exposure, and the downstream effects of systemic inflammation on PCa oncogenesis.

Nevertheless, these actionable findings do support the importance of structured and supervised lifestyle programs to reduce excess adiposity. Translating these findings into clinical utility with a lifestyle biomarker panel or molecular targeted therapies requires a validation study applying the same analysis to a different database (potentially with individual BMI details), investigating individual biomarker signatures and predictive power in a protein expression database, such as Olink and exploring drug links with PCa patients with ABCD, including the Glucagon‐Like Peptide‐1 (GLP‐1) receptor agonists group of drugs, using other bioinformatic tools. Interestingly, a recent study has also highlighted the use of GLP‐1 agonists in ABCD patients to reduce lifetime PCa risk relating to their influence on Forkhead Box C2 (FOXC2) gene expression and insulin resistance [91]. Ultimately, a clinical trial could be planned with examination of PCa tissue pre and post lifestyle and drug intervention, including for example, in vitro co‐culture of ppWAT and PCa cells with MCP‐1/IL‐1β inhibitors, or preclinical models of ABCD with microbiome manipulation. The development of immune panels in patients with ABCD as biomarkers of “cancer risk” which may be influenced by lifestyle changes is an enticing prospect. Further studies highlighted above may identify aspects of lifestyle and environmental factors to clarify what exposures in life are the best avoided to mitigate PCa risk.

The strengths of this study include a comprehensive review of the literature and the novel application of a network analysis to highlight metabolic drivers linking ABCD and PCa. This approach provides actionable strategies that can translate into clinical actions, as well as providing a basis for further research. There are important limitations, including the potential for publication and selection biases related to our literature search. Network analysis relies on the strength of the datasets used, and the TCGA database is based primarily on Western populations, so these findings may be less applicable to diverse ethnic groups. Both Network and GSEA may also result in interaction detection bias, partly mitigated by using a corrected *P* value to filter out false discovery interactions (see footnote in Table 2 for explanation).

## 5 Conclusion

Current knowledge of the metabolic drivers underlying the link between ABCD and PCa is critically reviewed, allowing conclusions regarding potential influences on PCa initiation, remodeling, and metastasis. Nevertheless, the heterogeneous nature of this evidence makes any deeper discovery as to their relative importance challenging. With this in mind, a network analysis was performed, demonstrating a core theme linking ABCD to immune cell activation, which promotes PCa development via MCP‐1, IL‐1β, and CXCL1 signaling. Top hypotheses to guide molecular targeted therapies and lifestyle biomarker panels for PCa in ABCD relate to such MCP‐1, IL‐1β, and CXCL1 signaling, as well as gut microbiome dysbiosis and exposure of the periprostatic white adipose tissue to the prostate microbiome. Further research and possibly clinical trials allowing histological examination of pre‐ and post‐lifestyle intervention PCa tissue may provide further insights.

## Author Contributions

**Jeffrey I. Mechanick:** conceptualization, writing – review and editing. **Elena Tomas Bort:** methodology, software, data curation. **Zachary Dovey:** conceptualization, writing – original draft, writing – review and editing.

## Funding

The authors have nothing to report.

## Disclosure

The authors have nothing to report. J.I.M. reports receiving honoraria from Abbott Nutrition and Merck for lectures and serves on the advisory boards of Abbott Nutrition, Aveta.Life, and Twin Health.

## Conflicts of Interest

The authors declare no conflicts of interest.

## Data Availability

Data sharing not applicable to this article as no datasets were generated or analyzed during the current study.
